# Soil Amendments and Slow-Release Urea Improved Growth, Physiological Characteristics, and Yield of Salt-Tolerant Rice Under Salt Stress Conditions

**DOI:** 10.3390/plants14040543

**Published:** 2025-02-10

**Authors:** Rongyi Li, Xiayu Guo, Yucheng Qi, Yuyuan Wang, Jianbo Wang, Pengfei Zhang, Shenghai Cheng, Wenli He, Tingcheng Zhao, Yusheng Li, Lin Li, Junchao Ji, Aibin He, Zhiyong Ai

**Affiliations:** 1 College of Breeding and Multiplication (Sanya Institute of Breeding and Multiplication), Hainan University, Sanya 572025, China; 18790676736@163.com (R.L.); 22220951310084@hainanu.edu.cn (Y.Q.); 16638535569@163.com (Y.W.); wangjianb1999@163.com (J.W.); 18738491817@163.com (P.Z.); csh17793665776@163.com (S.C.); hewenli43@163.com (W.H.); 17634021231@163.com (T.Z.); yushengli@hainanu.edu.cn (Y.L.); lilin18@hainanu.edu.cn (L.L.); 17331066936@163.com (J.J.); 2 National Innovation Center of Saline-Alkali Tolerant Rice in Sanya, Sanya 572000, China; wanilybao@163.com; 3 Hunan Hybrid Rice Research Center, Changsha 410125, China

**Keywords:** agronomic traits, antioxidant enzymes, nitrogen fertilizer, nitrogen metabolism, soil amendments, salt stress

## Abstract

The present study aimed to investigate the effects of different soil amendments coupled with nitrogen fertilizer on the morpho-physiological characteristics and yield of salt-tolerant rice under saline conditions. The soil amendments, i.e., S1: zeolite amendment, S2: coconut coir amendment, S3: humic acid amendment, and S0: no amendment, and fertilizer treatments, i.e., N1: urea, N2: slow-release urea, and N0: no N fertilizer, were kept in main plots and sub-plots, respectively, in a split-plot design. The salt-tolerant variety ‘Shuangliangyou 138’ was exposed to 0.3% salt irrigation water. The results showed that during the entire growth period, compared to S0, the S1 and S3 treatments increased the SPAD values by an average of 6.3%and 5.5%, respectively, the leaf area index by an average of 24.5% and 19.8%, the canopy interception rate by an average of 11.5% and 4.1%, and the aboveground biomass by an average of 36.8% and 13.9%, respectively. Moreover, under S1 and S3 conditions, the tiller number per square meter, leaf water potential, leaf water content, and chlorophyll contents were also improved under the slow-release urea than urea. Moreover, slow-release urea promoted root vitality and nutrient absorption as well as enhanced the activity of antioxidant and nitrogen metabolism enzymes than urea under the S1 and S3 conditions. In sum, the rational application of soil amendments and slow-release urea could improve the rice productivity on saline-alkali land.

## 1. Introduction

Rice is one of the major food crops whereas China is the largest producer and consumer of rice globally [1]. However, global climate warming and other external environmental factors are contributing to challenges such as soil salinization, which poses a serious threat to the secure production of food [2]; nevertheless, successful rice cultivation on saline-alkali lands can effectively contribute to food security [3].

Salt stress could have severe impacts on plants; for example, it causes a substantial reduction in leaf chlorophyll content [4], photosynthetic efficiency, leaf area index, and crop biomass [5]. Additionally, salt stress reduces tiller production, plant height, and panicle formation and grains per panicle, leading to a significant reduction in grain yields [6]. At the physiological level, salt stress causes metabolic imbalances in rice, induces membrane peroxidation, and results in physiological and biochemical damage [7]. Therefore, scientists have developed salt-tolerant rice varieties capable of maintaining yields under salt stress, offering a solution for boosting rice production in saline-alkali regions and enhancing food security. Previous studies have shown that salt-tolerant rice under mild salt stress maintains yield through a high chlorophyll content, photosynthetic efficiency, strong tillers, improved plant height, and improved panicle numbers and grains per panicle [8]. However, salt-tolerant rice experiences yield reductions when salt concentrations exceed 0.3% [9]. Therefore, it is imperative to adopt suitable agronomic cultivation measures to promote the growth and yield of salt-tolerant rice under severe saline conditions [10].

Soil amendments are convenient and effective agronomic cultivation measures that can effectively reduce the severe impacts of soil salinity on crop plants [11]. Currently, there are various types of soil amendments, such as gypsum, green manure, biochar, and organic silicon [9] which can improve soil structure and plant growth [12]. Moreover, humic acid can reduce membrane damage caused by salt stress and results in improved plant height, dry biomass, and resilience to salinity [13] whereas soil application of coconut coir as a substrate could also improve the plant root architecture under saline conditions. The research results indicate that compared to gypsum and biochar, zeolite can significantly increase the fresh weight of crops and promote the increase of root dry weight, thereby contributing to higher yields [14,15]. Meanwhile, relevant studies have found that compared to the application of gypsum, the use of humic acid can significantly increase the number of panicles and panicle length in rice, thereby further enhancing rice yield [16].

Nitrogen fertilization is a widely used agricultural practice to improve crop yields [17]. However, its impact on salinity-affected soils is complex and context-dependent [18,19]. While some studies have shown that nitrogen fertilization can improve plant growth under salt stress by enhancing nutrient uptake and photosynthetic efficiency [19], others have reported that improper use of nitrogen fertilizers may exacerbate salinity issues [20]. For example, excessive nitrogen application can lead to increased soil salinity through ion imbalances and reduced water infiltration [20]. Additionally, the effectiveness of nitrogen fertilization can vary depending on the type of fertilizer, application rate, soil type, and crop variety [20,21]

On the other hand, slow-release fertilizers can improve antioxidant enzyme activity in leaves and improve leaf physiological characteristics [22]. Additionally, slow-release N fertilizer can also improve rice quality, especially in terms of improving the structure of starch and enhancing the grain quality [23]. The combination of zeolite, humic acid, and slow-release urea offers unique synergistic benefits that have not been extensively explored in the context of saline-alkali soils for rice cultivation. Given the unique properties and synergistic benefits of zeolite, humic acid, and slow-release urea, our study aims to investigate their integrated effects on the growth, physiological characteristics, and yield of salt-tolerant rice under saline conditions. We hypothesize that this specific combination will significantly improve rice productivity by enhancing nutrient efficiency, improving antioxidant enzyme activity, and reducing the adverse effects of salinity stress.

## 2. Materials and Methods

### 2.1. Experimental Details and Crop Management

**Figure 1:**
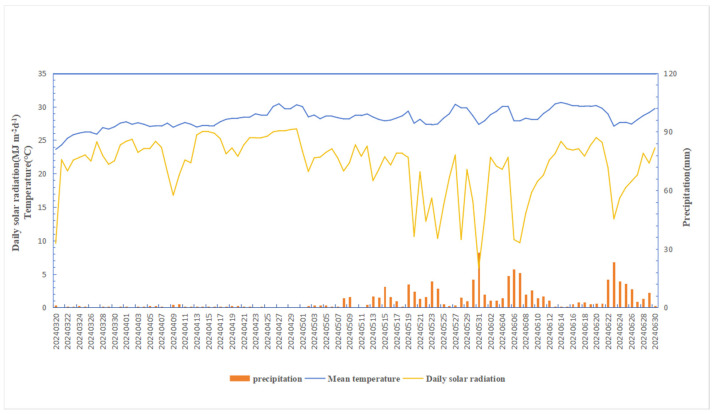
The temperature and rainfall during the experiment.

The experiment was conducted from April to August 2024 at the experimental base of the National Salt-Tolerance Rice Technology Innovation Center, Leyi Village, Hainan Province, China (108.90′ E, 18.44′ N). The average daily temperature throughout the entire growth period was 28.2 °C, and the total rainfall was 380.7 mm. The average daily solar radiation was 21.3 MJ m^−2^d^−1^ (Figure 1). The initial soil salinity level was around 0.2%. The basic physicochemical properties of the soil were as follows: total nitrogen 0.3 g kg ^−1^, available nitrogen 78.5 mg kg^−1^, total potassium 6.2 g kg^−1^, and available potassium 78.5 mg kg^−1^. The seedlings salt-tolerant rice variety ‘Shuangliangyou 138′ was transplanted at the three-leaf and one-heart stage with a 20 × 20 cm row spacing. The fertilizer treatments, i.e., N1: urea, N2: slow-release urea, and N0: no N fertilizer, and soil amendments, i.e., S1: zeolite amendment, S2: coconut coir amendment, S3: humic acid amendment, and S0: no amendment, were kept in main plots and sub-plots, respectively, in a split-plot design. The structural properties of the soil amendments provided for the test were as follows: zeolite: SiO_2_ = 67.1%; Al_3_O_3_ = 11.8%; moisture = 8.5% (supplied by Hebei Shijiazhuang JingSen Mineral Co., Ltd., Shijiazhuang, China); humic acid with free humic acid on dry basis = 51.4%; moisture = 7.7%; pH: 10.3 (supplied by Inner Mongolia Delang Biological Co., Inner Mongolia, Ltd., Inner Mongolia, China); coconut coir: pH = 5.1; total N: 7.1%; total phosphorus: 0.3%; total potassium: 3.4% (supplied by Sanya Sanliyuan Coconut Coir Company, Sanya, China). The fertilizers used were slow-release urea and conventional urea with a N content of 44 and 46%, respectively. The slow-release urea was resin-coated slow-release urea and the slow-release period was 90 days.

The experimental plots were surrounded by a 20 cm soil ridge around each subplot with an area of 20 m^2^. The soil amendments were applied before rice planting, i.e., the substrates were spread in the field and mixed in the soil with a soil cultivator uniformly. The phosphatic fertilizer was applied at 60 kg hm^−2^ (one-time base fertilizer) and KCl at 100 kg hm^−2^ (applied in a 1:1 ratio of basal fertilizer and panicle fertilizer). The total amount of N for the entire growth period was 150 kg hm^−2^, with 80% as the basal dose (applied 3–5 days after rice seedling transplanting) and 20% at the panicle stage (applied 15–20 days after flowering).

To simulate salt stress, a saline pond was constructed around the experimental field with a mixture of seawater and freshwater, maintaining a salinity of approximately 0.3%. Saline irrigation was initiated after the rice seedlings recovered from transplanting, and the soil salinity was monitored daily using a portable conductivity meter (2266FS, Spectrum, Boston, MA, USA) to ensure that the field salinity remained at 0.3%. A water layer of about 5 cm was maintained throughout the growth period, and water was cut off 7 days before harvest. Pest, disease, and weed control was carried out in accordance with unified rice cultivation measures.

### 2.2. Determination of Agronomic Traits

At the mid-tillering stage (MT), panicle initiation stage (PI), heading stage (HS), and maturity stage (MS), six uniformly growing plants were randomly selected from each subplot and the plant height and tiller numbers and dry biomass were measured. The bleeding intensity measurement was determined by Chen et al. [24]

### 2.3. Rice Leaf SPAD Value (Chlorophyll Content) and Canopy Intercept Rate

The SPAD value was measured via a portable chlorophyll meter during the whole growth period. From each replicate, four uniform plants were selected, and fully expanded leaves were measured during the MT stage. After the PI, sword leaves were measured three times at the top, middle, and bottom, and the average value was taken. At the MT, PI, and HS of rice, the cumulative incident light and light interception of the canopy were measured via a canopy analyzer (AccuPAR LP-80, Decagon Devices Inc., Pullman, WA, USA) to calculate the light interception rate (LI%).

### 2.4. Determination of Chlorophyll Contents After HS

After HS, the samples were taken from the field every five days. Fresh rice leaves (0.1 g) were cut into small pieces, and extracted with 20 mL of anhydrous ethanol for 48 h. The absorbance at wavelengths of 663, 645, and 470 nm was measured via a UV-4802 spectrophotometer (Shimadzu Corporation, Kyoto, Japan) and the contents of Chl a, Chl b, and Chlorophyll a + b were estimated according to Li et al. [25].

### 2.5. Leaf Water Content and Leaf Area Indices

Five fully expanded leaves were taken from each subplot, fresh weight was taken as ‘F1’, and then dried in a 75 °C oven until a constant weight ‘F2’. The water content of the leaves was estimated as F1 – F2. From each subplot, eight representative plants were selected, the green leaves and sheaths were separated, and the length and width were measured and multiplied with a factor of 0.8 to calculate the sample leaf area. The leaf samples were dried in a 75 °C oven to a constant weight and then to calculate the leaf area index.

### 2.6. Measurement Antioxidant and N Metabolic Enzyme Activities

At the MT, PI, and HS, the uppermost rice leaves from each treatment were cut, immersed in liquid N_2_ and stored at −80 °C. The antioxidant and N metabolic enzyme activities were measured according to the operating instructions of the kit obtained by Beijing Solei Bao Technology Co., Ltd. (Beijing, China).

### 2.7. Yield and Yield Component Measurements

At MS, three survey points were selected in each experimental subplot, and 20 effective panicles were surveyed at each point. Then, five uniform panicles were taken as one sample from each experimental subplot, and the total number of grains per panicle, filled grain rate, and 1000-grain weight were measured. In each subplot, an area of 3 m^2^ was harvested and the yield was calculated at 13.5% grain moisture contents.

Data were organized using Microsoft Excel 2016 (Microsoft Inc., Redmond, WA, USA). Two-factor analysis of variance (ANOVA) was performed using SPSS 19.0 software (SPSS Inc., Chicago, IL, USA). Differences among treatments were separated using Tukey’s post hoc test at a significance level of 0.05. All figures were created using Origin 9.0 software (OriginLab Corporation, Northampton, MA, USA).

## 3. Results

### 3.1. Yield and Yield Components

Soil amendments in integration with N fertilizers substantially affected the yield and yield attributes of rice under saline conditions (Table 1). Among the soil amendments, the S1 had the highest yield, i.e., 14.5%, 7.9%, and 18.2% higher than S2, S3, and S0, respectively. For the number of grains per panicle and effective panicles, the soil amendments were ranked from highest to lowest as follows: S1 > S3 > S2 > S0. Furthermore, the S1 increased the number of spikelets per panicle by 7.3%, 3.4%, and 8.7%, as compared with S2, S3, and S0, respectively. However, there were no significant differences in the 1000-grain weight or filled grain rate among the soil amendments.

In addition, the grain yield in N1 and N2 were significantly higher than N0 under the soil amendment treatments (Table 1). Moreover, under S1 conditions, the grain yield in N2 was increased by 10.8% and 33.1% than N1 and N0, respectively. Compared with N1 and N0, the N2 increased the grain yield by 5.0% and 34.4%, respectively, under S3. The N2 treatment had the greatest effect on the number of spikelets per panicle under the S1, S3, and S0 conditions. For the panicles per m^2^, under the S1, S2, and S3 conditions, there were significant differences among the N fertilizer treatments, and they were ranked from highest to lowest as follows: N2 > N1 > N0. Compared with N0, the N1 led to an increase of 4.0%, with no significant differences among the N fertilizer treatments under S2.

### 3.2. Tiller Number, Plant Height, Biomass, and Leaf Area Index

**Figure 2:**
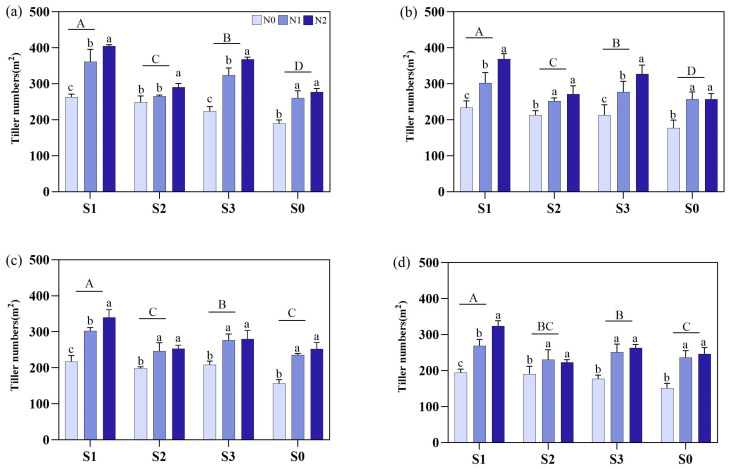
Effects of different soil amendments and different nitrogen sources on tiller square meters in different growth stages under salt stress. (**a**) represents the mid-tillering stage, (**b**) represents the panicle initiation stage, (**c**) represents the heading stage, and (**d**) represents the mature stage. N1: urea, N2: slow-release urea, N0: no nitrogen fertilizer; S1: zeolite amendment, S2: coconut coir amendment, S3: humic acid amendment, S0: no amendment. Different lowercase letters indicate significant differences between different nitrogen fertilizers under the same soil amendment, and different uppercase letters indicate significant differences among different soil amendments.

Different soil amendments and N fertilizer types significantly affected the tillers m^2^, whereas the S1 treatment resulted in the greatest number of tillers. Additionally, during the whole growth period, the tillers m^2^ among the soil amendments was ranked from highest to lowest as S1 > S3 > S2 > S0. The tillers m^2^ were enhanced by 12.4–41.4% during the MT, 10.7–23.6% during the PI, 12.4–33.4% during the HS, and 13.8–24.3% during the MS in S1 than S2, S3, and S0, respectively (Figure 2).

For the different N fertilizer types, during the MT under S1, S2, and S3, N2 significantly increased by 37.3%, 6.9%, and 45.3%, respectively, compared with N0. During the PI, the N2 significantly increased tillers m^2^ under S1 and S3 conditions by 58.4% and 53.5%, respectively, compared to N0, and by 22.2% and 18.1% compared to N1. During the HS, the N2 increased tillers m^2^ under S1 by 55.9% and 12.6% compared to N0 and N1, respectively, whereas N2 also increased the tillers m^2^ by 27.8%, 34.6%, and 51.3% under S2, S3, and S0, respectively (Figure 2).

**Figure 3:**
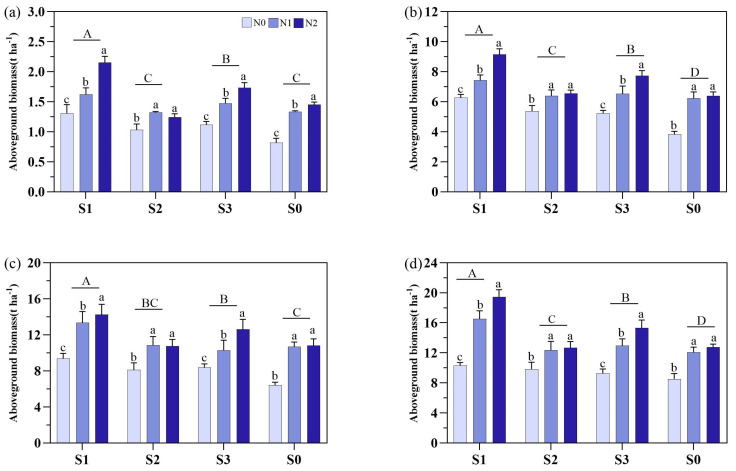
Effects of based different soil amendments of different nitrogen sources on aboveground biomass in different growth stages under salt stress. Notes: (**a**) represents the mid-tillering stage, (**b**) represents the panicle initiation stage, (**c**) represents the heading stage, and (**d**) represents the mature stage. N1: urea, N2: slow-release urea, N0: no nitrogen fertilizer; S1: zeolite amendment, S2: coconut coir amendment, S3: humic acid amendment, S0: no amendment. Different lowercase letters indicate significant differences between different nitrogen fertilizers under the same soil amendment, and different uppercase letters indicate significant differences among different soil amendments.

Soil amendments and N fertilizers significantly influenced dry biomass across growth stages (Figure 3). The S1 treatment resulted in the highest aboveground biomass which was 17.4–42.0% higher than S0 at MT, 17.1–38.9% at PI, 24.7–32.6% at HS, and 9.9–23.2% at MS. For N fertilizers, the N2 resulted in the highest dry biomass under S1 and S3 conditions with the following trend of N2 > N1 > N0. Moreover, no significant differences were observed between N2 and N1 under the S2 and S0 treatments, except during MT under S0.

Different N fertilizer types and soil amendments substantially affected the plant height during different growth stages (Table 2). Moreover, during the PI and HS, there were significant interactive effects between N fertilizer types and soil amendments. During MT and PI, the plant height among the treatments was the highest in the S1 treatment with no significant difference between the S2 and S0 treatments. During the HS, S1, S2, and S3 were all significantly greater than S0, with an increase of 4.2–4.6%. Additionally, at PI, under various soil amendments, the plant height in N1 and N2 treatments did not differ significantly (except for the PI under S3), but both were significantly greater than N0.

Furthermore, different N fertilizer types and soil amendments significantly affected the leaf area index (LAI) across growth stages (Table 3). During MT, the S1 increased LAI by 54.4%, 44.4%, and 46.2% compared to S2, S3, and S0, respectively. At PI and HS, the S1 had the highest LAI, trending as S1 > S3 > S2 > S0, with significant differences amongst treatments. For N fertilizers, the LAI generally followed the trend N2 > N1 > N0 across growth stages (except for MT and HS under S0, respectively). During PI and HS (excluding HS under S0), the N2 caused a significant effect on leaf area than N1 under all soil amendments.

### 3.3. SPAD Values, Chlorophyll Contents After HS, and Canopy Intercept Rate

Different N fertilizer types and soil amendments significantly affected chlorophyll content during different growth stages (Table 4). During MT, chlorophyll content was increased by 7.8%, 3.6%, and 5.5% in S1, S2, and S3, respectively, compared to S0. At PI, S1 had the highest SPAD values, increased by 2.5% and 6.5% compared to S2 and S0, with no significant difference between S1 and S3. At HS, the chlorophyll content in S1, S2, and S3 were statistically similar, showing an increase of 4.8%, 4.5%, and 3.9%, compared to S0, respectively. For N fertilizers, during MT, the chlorophyll contents in N1 and N2 under the S1, S3, and S0 treatments was significantly higher than N0. At PI, the N1 led to an increase of 7.1%, 6.5%, and 14.1%, and N2 by 11.0%, 4.3%, and 11.8% than N0 in chlorophyll contents under S1, S2, and S3, respectively. At HS, the chlorophyll contents in N2 were 14.0%, 4.4%, and 9.7% higher than N0 under S1, S2, and S3, respectively

**Figure 4:**
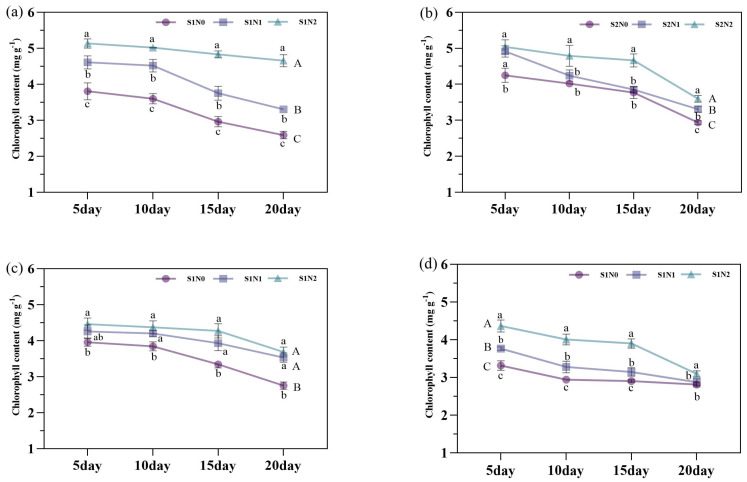
Effects of different soil amendments and different nitrogen sources on chlorophyll at 5 days, 10 days, 15 days, and 20 days after the heading stage under salt stress. Notes: (**a**) represents zeolite amendment, (**b**) represents coconut coir amendment, (**c**) represents humic acid amendment, and (**d**) represents no amendment. N1: urea, N2: slow-release urea, N0: no nitrogen fertilizer; S1: zeolite amendment, S2: coconut coir amendment, S3: humic acid amendment, S0: no amendment. Different lowercase letters indicate significant differences among different nitrogen fertilizers at the same time for the same amendment, and different uppercase letters indicate significant differences over all times for the same amendment with different nitrogen fertilizers.

Figure 4 showed the dynamic trend of chlorophyll degradation after heading in rice, with chlorophyll content decreasing as growth progresses. Under S1, N2 significantly increased the chlorophyll content by 11.0–41.0%, compared to N1 and by 34.1–80.0% compared to N0. The N1 also increased chlorophyll content by 21.2–27.7% compared to N0. Under S3, the chlorophyll contents in N1 and N2 was increased by 7.7–28.4% and 4.4–28.0%, respectively, compared to N0.

Additionally, N fertilizers and soil amendments significantly affected the canopy interception rate, with notable interactions during the PI and HS stages (Table 5). During MT, PI, and HS, the S1 increased the canopy interception rate by 8.8–21.9% and 7.1–26.7% compared to S2 and S0, respectively. For N fertilizers, during MT, N1 and N2 under the S1, S2, S3, and S0 treatments were similar but significantly higher than N0. During PI and HS under S1, the interception rate in N2 was increased by 22.9% and 55.4%, and N1 by 9.1% and 48.7%, compared to N0. Under S2, the interception rate in N2 was increased by 22.9% and 12.1% compared to N0 whereas under S3 and S0, the following trend was noted regarding interception rate: N2 > N1 > N0.

### 3.4. Leaf Water Content and Root Bleeding Intensity

Different N fertilizer types and soil amendments significantly affected the leaf water content during various stages (Table 6). At MT and HS, the S1 increased leaf water contents by 1.7–2.0% and 1.6–2.2% compared to S2, S3, and S0. At PI, S3 had the highest leaf water contents, significantly exceeding other treatments, while there were no differences among S1, S2, and S0. For N fertilizers at MT under S2, N1 increased the leaf water contents by 3.7% and 3.7% compared to N2 and N0. At PI, it was N2 > N1 > N0 under S2, S3, and S0. At HS, N2 and N1 were significantly higher than N0 under S2.

**Figure 5:**
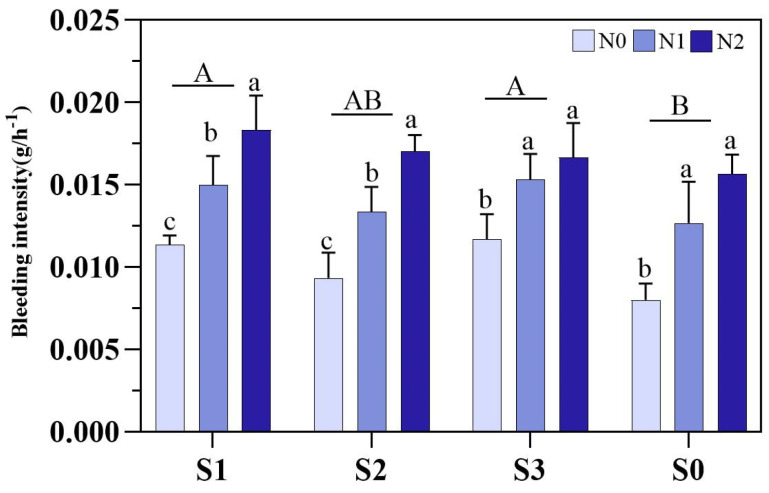
Effects of different soil amendments and different nitrogen sources contents on bleeding intensity at different growth stages under salt stress. N1: ordinary urea, N2: slow-release urea, N0: no nitrogen fertilizer; S1: zeolite amendment, S2: coconut coir amendment, S3: humic acid amendment, S0: no amendment. Different lowercase letters indicate significant differences among different nitrogen fertilizers at the same time for the same amendment, and different uppercase letters indicate significant differences over all times for the same amendment with different nitrogen fertilizers.

Moreover, at HS, the S1 and S3 increased root bleeding by 23.1% and 20.7%, respectively, compared to S0. For N fertilizers, under S1 and S2, the following trend was noticed regarding root bleeding: N2 > N1 > N0. In addition, under S3 and S0, the N1 and N2 were significantly higher than N0 (Figure 5).

### 3.5. Antioxidant Enzyme Activity and Malondialdehyde (MDA) Content

**Figure 6:**
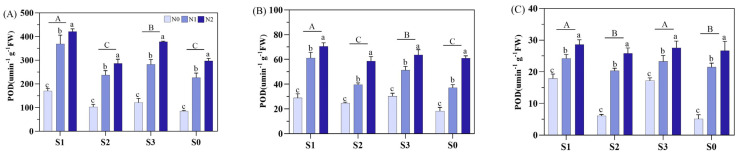
Effects of different soil amendments and different nitrogen sources on peroxidase (POD) at different growth stages under salt stress. Notes: POD: (**A**) represents the mid-tillering stage, (**B**) represents the panicle initiation stage, and (**C**) represents the heading stage. N1: ordinary urea, N2: slow-release urea, N0: no nitrogen fertilizer; S1: zeolite amendment, S2: coconut coir amendment, S3: humic acid amendment, S0: no amendment. Different lowercase letters indicate significant differences between different nitrogen fertilizers for the same soil amendment, and different uppercase letters indicate significant differences between different soil amendments for the same nitrogen fertilizer.

**Figure 7:**
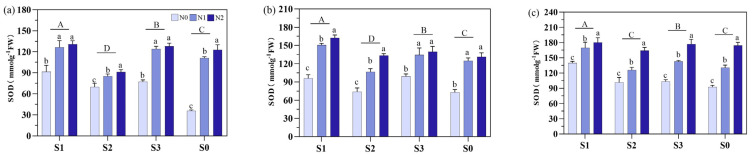
Effects of different soil amendments and different nitrogen sources on superoxide dismutase (SOD) activities at different growth stages under salt stress. Notes: SOD: (**a**) represents the mid-tillering stage, (**b**) represents the panicle initiation stage, and (**c**) represents the heading stage. N1: ordinary urea, N2: slow-release urea, N0: no nitrogen fertilizer; S1: zeolite amendment, S2: coconut coir amendment, S3: humic acid amendment, S0: no amendment. Different lowercase letters indicate significant differences between different nitrogen fertilizers for the same soil amendment, and different uppercase letters indicate significant differences between different soil amendments for the same nitrogen fertilizer.

Soil amendments and N fertilizer types significantly influenced peroxidase (POD) and superoxide dismutase (SOD) activities during various stages (Figure 6 and Figure 7). The S1 increased the POD activity at MT by 53.2%, 22.9%, and 57.2% compared to S2, S3, and S0, respectively, and at PI by 30.6%, 10.6%, and 37.9%. At HS, the S1 and S3 showed no significant difference but exceeded S2 and S0. Overall, soil amendments ranked as S1 > S3 > S2 > S0 regarding POD activity. Similarly, the N fertilizer treatments were ranked as N2 > N1 > N0 at MT, PI, and HS for POD activity with significant differences among treatments.

Regarding SOD activity, S1 had the highest SOD activity throughout the growth period and ranked as S1 > S3 > S2 > S0 (Figure 7). At MT, S1 increased the SOD activity by 41.6%, 5.9%, and 29.4% compared to S2, S3, and S0, respectively, whereas the SOD activity was increased by 30.1%, 9.5%, and 24.3% (at PI) and 25.2%, 15.7%, and 23.4% (at HS). For N fertilizers, S1 and S3 showed no difference between N1 and N2 at MT and PI but were substantially higher than N0. At PI, N2 increased the SOD activity by 69.4–80.2% across soil amendment treatments, and N1 by 41.0–88.3%. At HS, the N treatments followed the following trend regarding SOD activity: N2 > N1 > N0, with significant differences among treatments.

**Figure 8:**
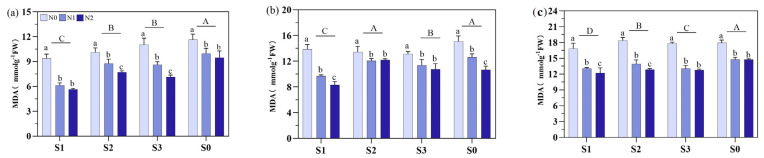
Effects of different soil amendments and different nitrogen sources on the malondialdehyde (MDA) content at different growth stages under salt stress. Notes: (**a**) represents the mid-tillering stage, (**b**) represents the panicle initiation stage, and (**c**) represents the heading stage. N1: ordinary urea, N2: slow-release urea, N0: no nitrogen fertilizer; S1: zeolite amendment, S2: coconut coir amendment, S3: humic acid amendment, S0: no amendment. Different lowercase letters indicate significant differences between different nitrogen fertilizers within the same soil amendment, and different uppercase letters indicate significant differences between different soil amendments.

Different soil amendments and N fertilizer types significantly affected the MDA content during the various growth periods (Figure 8). At MT, the MDA contents in S1, S2, and S3 were decreased by 25.8%, 16.7%, and 15.9%, respectively, compared with S0 whereas no significant difference between the S0 and S2 treatments were noticed at PI, but both were significantly higher than those in the S1 and S3 treatments. Regarding N fertilizer, from MT to HS, the N1 and N2 treatments under soil amendments substantially reduced the MDA contents than N0.

### 3.6. Leaf N Metabolic Enzyme Activity

**Figure 9:**
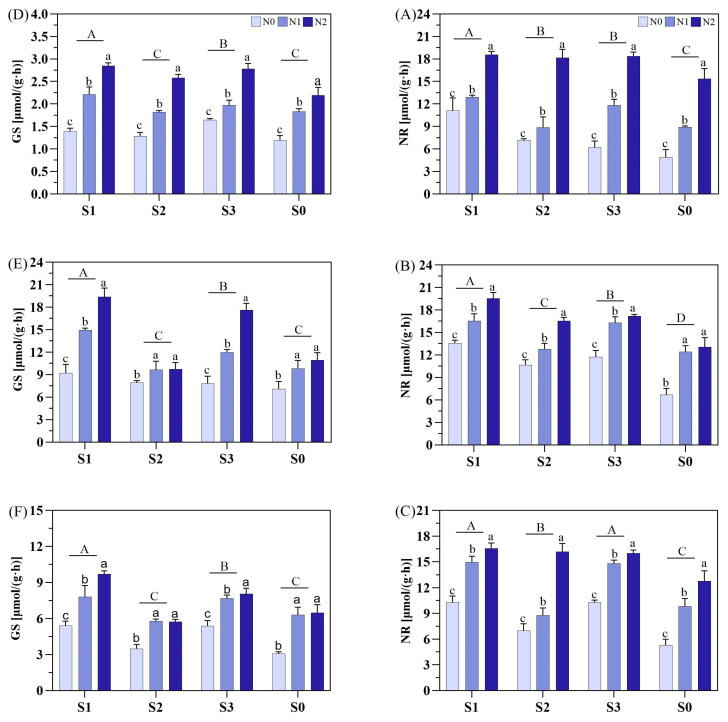
Effects of different soil amendments and different nitrogen sources on nitrate reductase (GS) and nitrate reductase (NR) in different growth stages under salt stress. Notes: GS: (**D**) represents the mid-tillering stage, (**E**) represents the panicle initiation stage, (**F**) represents the heading stage. NR: (**A**) represents the mid-tillering stage, (**B**) represents the panicle initiation stage, (**C**) represents the heading stage. N1: ordinary urea, N2: slow-release urea, N0: no nitrogen fertilizer; S1: zeolite amendment, S2: coconut coir amendment, S3: humic acid amendment, S0: no amendment. Different lowercase letters indicate significant differences between different nitrogen fertilizers within the same soil amendment, and different uppercase letters indicate significant differences between different soil amendments.

Soil amendments and N fertilizer types significantly influenced glutamine synthetase (GS) and nitrate reductase (NR) activities during various stages, with S1 consistently having the greatest effect (Figure 9). Regarding GS activity, S1 had the highest GS activity throughout the growth period, significantly exceeding other treatments whereas the GS activity for S3 was higher than S2 and S0. For N fertilizers, GS activity ranked as N2 > N1 > N0 under all soil amendments at MT. However, under S1 and S3, N2 showed the highest GS activity during PI, increased by 29.7% and 110.5% (in S1) and 47.2% and 125.0% (in S3) compared to N1 and N0, respectively. Under S2 and S0, N1 and N2 had no significant differences but the GS activity was higher than N0. Similar trends were also observed at PI and HS under saline conditions.

For NR activity, S1 had the highest NR activity throughout the growth period. S1 increased NR by 24.5%, 17.0%, and 45.9% (at MT) and 24.3%, 9.8%, and 54.4% (at PI), as compared to S2, S3, and S0, respectively. On the other hand, the S1 and S3 showed no statistical differences but were significantly higher than S2 and S0 at HS. Likewise, for N fertilizers, the NR activity was ranked as N2 > N1 > N0 under all soil amendments.

## 4. Discussion

### 4.1. Impact of Soil Amendments and N Fertilizers on Rice Yield

In previous studies, various ways in which salt stress inhibits plant growth and yield have been reported. For instance, salt stress leads to a decrease in pollen viability, resulting in the production of empty grains in rice, which is one of the key factors leading to yield reduction [26]. The lack of nutrients caused by salt stress disturbs source–sink relationships which in turn affects the development grain formation mechanism [27]. The present study indicated that zeolite and humic acid amendments significantly improved the rice yield under salt stress conditions owing to an improved number of effective panicles and grains per panicle (Table 1). Previously, Wu et al. [28] reported that zeolite and N fertilizer resulted in efficient nutrient uptake which steadily maintained the rice filling period and filling rate and significantly increased the number of effective panicles and grains per panicle compared with N fertilizer without zeolite treatment. The zeolite amendments improved the N use efficiency, water use efficiency, and yield of rice. Compared with those in the treatments without zeolite amendments, the number of effective panicles and grains per panicle significantly increased [29]. Moreover, application of humic acid resulted in greater rice panicle lengths and effective panicle numbers and improved rice yield under Cd-contaminated conditions [30]. Similarly, Shaaban et al. [31] reported that the application of humic acid amendments in saline-alkali soil improved the soil and maintained agronomic traits of rice.

The application of N fertilizer is also closely related to the formation of the rice sink and yield factors under salt stress. In this study, under the zeolite and humic acid conditions, the slow-release N fertilizer significantly increased the rice yield owing to an improvement in the number of effective panicles and grains per panicle, compared to urea (Table 1). Under the conditions of S1 and S3, the yield with slow-release urea fertilizer was higher than that with urea, mainly because the slow-release urea fertilizer can gradually release nitrogen according to the plant’s needs, reducing nitrogen loss and improving nitrogen fertilizer use efficiency [32,33]. Urea is a quick-release nitrogen fertilizer with a rapid nitrogen release rate. In the early stages of fertilization, urea quickly decomposes into ammonium and nitrate nitrogen. If these nitrogen compounds are not promptly absorbed by plants, they can easily be lost through volatilization and leaching [34]. Although the rapid-release characteristic of urea can quickly increase soil nitrogen content in the initial stage of fertilization, the insufficient nitrogen supply in the later stages may lead to nutrient deficiency in plants during the mid-to-late growth periods, thereby affecting photosynthesis and biomass accumulation [35].

### 4.2. Impact of Soil Amendments and N Fertilizers on Rice Growth

In this study, the combination of zeolite, humic acid, and slow-release N fertilizer significantly increased tillering, biomass, and plant height and further improved the leaf area index (Table 2 and Table 3, Figure 1 and Figure 2). The results were in line with Wu et al. [36], who reported that the slow-release characteristics of zeolite, when used in combination with N fertilizer, increased the N use efficiency, leaf area index, and aboveground dry matter of crops, and significantly retained the nutrient supply for rice and final yield. Specifically, zeolite and humic acid as soil amendments not only improve soil physical properties but also reduce the negative impact of salt stress on rice. Furthermore, the use of slow-release urea not only reduces N loss but also increases the efficiency of N utilization, which is crucial for improving rice photosynthesis and biomass accumulation [37,38,39]. Moreover, the present study found that the zeolite treatment significantly improved the bleeding intensity and SPAD values before heading (Table 4 and Figure 4), which is consistent with the findings of Sun et al. [40] who reported that the addition of zeolite amendments increased the degree of root bleeding, improved the leaf SPAD values, and further increased the aboveground dry matter content [40]. Likewise, Malekian et al. [41] reported that the application of zeolite and modified zeolite resulted in the maximum leaf area index, dry matter accumulation, N absorption, and grain yield. In saline-alkali soils, poor soil structure and weak fertility, along with low ion adsorption capacity, accelerate the loss of ordinary urea. Although zeolite acts as a slow-release carrier, it cannot prevent nutrient loss from the urea, leading to N deficiency. In contrast, the combination of slow-release N fertilizer and zeolite ensures more efficient N utilization, providing a stable supply of nutrients that support crop growth, enhance stress resilience, and lead to differences in chlorophyll content later. Tahir et al. [42] reported that the application of humic acid increased plant height and plant biomass, and promoted crop growth and development [42] whereas application of humic acid can maintain photosynthesis under stress, delay crop aging, reduce leaf water loss, and maintain normal crop growth and development [43]. In this study, especially during the PI, the leaf water content in the humic acid treatment was significantly greater than that in the treatment without amendments (Table 6). Application of humic acid is a common method used to improve crop growth and soil fertility. Previous studies have shown that the addition of relatively high proportions of humic acid significantly increase the leaf area index, biomass, and plant height of rice [44].

### 4.3. Impact of Soil Amendments and N Fertilizers on Rice Antioxidant Enzyme Activity

In this study, compared with no amendment, humic acid amendment significantly increased POD and SOD activity and significantly reduced the MDA content during the MT, PI, and HS (Figure 5 and Figure 6). The results were consistent with the findings of Rao et al. [45], who reported that the combined or separate application of silicate and humic acid significantly increased antioxidant enzyme activity and reduced the MDA content in salt-stressed seedlings. Salt stress is an abiotic stress that harms plant growth and development and affects plants in four main ways, i.e., osmotic stress, nutritional stress, ionic stress, and the accumulation of reactive oxygen species (ROS). When excessive ROS accumulate, plants activate their ROS-scavenging mechanisms, including the enhancement of enzyme activities such as POD and SOD [46]. Under salt stress conditions, the soil amendments and N fertilizer treatments significantly increased the activity of antioxidant enzymes, including POD and SOD, in rice leaves. The increase in antioxidant enzymes help to alleviate the oxidative damage caused by salt stress and protects rice from oxidative damage [47]. The application of zeolite particles under salt stress can increase antioxidant capacity, maintain water and nutrient retention, and related physiological mechanisms [48]. Mahmoud et al. [49] reported that, under stress conditions, the application of zeolite increased a water use efficiency and decreased transpiration rate [49]. Moreover, humic acid significantly reduced the plant MDA content and increased the corresponding antioxidant enzyme activity, further enhancing plant stress resistance. Under salt stress conditions, humic acid plays an important role in maintaining ion homeostasis, regulating appropriate osmotic content, and constructing an effective antioxidant defense system, and it has the potential to increase crop salt tolerance [50].

### 4.4. Impact of Soil Amendments and N Fertilizers on Rice N Metabolism

The application of soil amendments and N fertilizers significantly affected the activities of glutamine synthetase (GS) and nitrate reductase (NR) enzymes, which play key roles in N metabolism, in rice leaves. For example, the application of zeolite and humic acid significantly increased the activity of the enzymes GS and NR, helping to maintain rice N assimilation under salt stress conditions (Figure 7). The combined application of zeolite and N fertilizer significantly increased the water and nutrient retention rates but also significantly increased the accumulation of N, increasing the activity of N metabolic enzymes within the plant [40]. Pisarović et al. [51] reported that the application of zeolite to sandy loam and dust soils inhibited the conversion of NH_4_^+^ to NO_3_^−^. When zeolite was mixed with N fertilizer, N loss was reduced by 19–22% through leaching and volatilization. The addition of humic acid to nano-N fertilizer and conventional urea N fertilizer resulted in the highest availability, promoting the efficient absorption and utilization of N by crops [52]. Kong et al. [53] found that compared with the sole application of urea, humic acid urea reduced fertilizer N loss by 25.51% and 23.07% in terms of N leaching, NH_3_ volatilization, and N_2_O emissions, respectively. Humic acid combined with urea enhanced soil N availability, alleviated microbial nutrient limitations, and improved soil quality. This combination increased crop yield and N fertilizer use efficiency by reducing N loss and extending fertilization effects [53]. In this study, humic acid combined with slow-release N fertilizer significantly increased the activity of GS and NR compared with zeolite combined with ordinary urea, indicating that the combination of humic acid and slow-release urea can maintain a good N metabolism level in rice. The complex relationship between plant growth and abiotic stress depends on N metabolism. Under salt stress conditions, there is an antagonistic and synergistic effect between salinity and N levels. The consumption of chlorophyll in plants induced by salinity can be alleviated by optimal N supplementation; however, excessive N can exacerbate salt stress and negatively affect plant growth and development [54]. Moreover, under different soil amendments, the application of ordinary urea and slow-release urea further increased the N metabolism level in rice, especially the combined application of zeolite and slow-release urea, as slow-release urea gradually dissolves, part of it is absorbed and used by crops, while the remaining NH^4+^ is adsorbed by the zeolite [55], reducing N fertilizer loss. When soil N becomes insufficient, the adsorbed N fertilizer is released, sustaining the N supply, ensuring adequate nutrition under salt stress, and supporting normal rice growth and development.

## 5. Conclusions

In summary, applying soil amendments and N fertilizers improved rice growth and yield, primarily by increasing the number of effective panicles and grains per panicle. Soil amendments and slow-release N fertilizer enhanced the tillering, biomass, plant height, chlorophyll content, and leaf area index, while reducing malondialdehyde content and boosting antioxidant enzyme activity, thus improving stress resistance. Zeolite and humic acid, as soil amendments, helped to maintain N metabolism, supporting enzyme activity and improving N utilization of rice under salt stress. Overall, combining soil amendments and slow-release fertilizers can effectively improve rice growth and yield under salt stress, with zeolite and humic acid offering practical solutions for food security. In summary, the integration of slow-release urea with soil amendments like zeolite and humic acid offers a practical, commercially accessible, and sustainable solution for improving rice productivity on saline-alkali lands. By adopting these strategies, farmers can enhance their yields and contribute to sustainable agricultural practices. While our study provides valuable insights into the effectiveness of specific soil amendments for mitigating salinity stress in rice, future research should focus on addressing regional variability, crop-specific responses, and long-term impacts. By developing tailored strategies and integrating these findings with broader agricultural management practices, the potential benefits of soil amendments can be realized across diverse crops and geographic regions. This approach will be crucial for enhancing agricultural productivity and ensuring food security in saline-affected areas.

## Figures and Tables

**Table 1:** Effects of different types of soil amendments and nitrogen sources on rice yield and yield attributes at different growth stages.

Soil Amendments	Nitrogen	Spikelets Panicle^−1^	Panicles (no.m^2^)	Filled Grain Rate (%)	1000-Grain Weight (g)	Grain Yield (t ha^−1^)
S1	N1	192.7 ± 6.7b	226.0 ± 4.0b	84.3 ± 3.8a	20.7 ± 0.1a	6.0 ± 0.1b
	N2	210.7 ± 5.9a	265.9 ± 5.3a	85.0 ± 5.6a	20.5 ± 0.3a	6.7 ± 0.2a
	N0	178.7 ± 2.3c	175.0 ± 5.0c	87.7 ± 5.0a	20.5 ± 0.3a	5.1 ± 0.1c
	Mean	194.0 ± 4.7A	222.3 ± 1.4A	85.7 ± 1.5A	20.5 ± 0.2A	5.9 ± 0.1A
S2	N1	200.3 ± 5.5a	209.7 ± 8.1b	84.0 ± 4.4a	20.5 ± 0.5a	5.6 ± 0.1a
	N2	180.7 ± 3.5b	222.7 ± 7.0a	82.7 ± 3.5a	20.2 ± 0.3ab	5.7 ± 0.1a
	N0	161.3 ± 1.5c	166.9 ± 6.2c	86.7 ± 7.0a	19.8 ± 0.1b	4.2 ± 0.1b
	Mean	180.8 ± 1.7C	199.8 ± 6.6C	84.5 ± 4.5A	20.4 ± 0.2A	5.2 ± 0.1C
S3	N1	198.3 ± 3.8a	218.7 ± 2.9b	85.3 ± 5.0a	20.5 ± 0.3a	5.8 ± 0.2b
	N2	201.7 ± 8.1a	233.8 ± 3.0a	80.7 ± 2.1a	20.6 ± 0.5a	6.1 ± 0.1a
	N0	163.0 ± 8.9b	177.1 ± 4.1c	85.3 ± 5.0a	20.1 ± 0.5a	4.5 ± 0.2c
	Mean	187.7 ± 6.9B	209.9 ± 0.6B	83.8 ± 2.7A	20.4 ± 0.7A	5.5 ± 0.1B
S0	N1	187.0 ± 4.4a	221.3 ± 3.2a	87.0 ± 2.0a	20.7 ± 0.3a	5.7 ± 0.2a
	N2	191.3 ± 4.5a	219.8 ± 7.8a	86.0 ± 3.6a	20.4 ± 0.5a	5.8 ± 0.1a
	N0	157.0 ± 7.0b	143.3 ± 8.1b	87.3 ± 2.1a	20.1 ± 0.1a	3.5 ± 0.2b
	Mean	178.4 ± 3.6C	194.8 ± 2.8C	86.8 ± 0.8A	20.4 ± 0.2A	5.0 ± 0.1D
ANOVA	S	*	**	ns	ns	***
	N	***	***	ns	*	***
	S × N	***	***	ns	ns	***

Notes: N1: urea, N2: slow-release urea, N0: no nitrogen fertilizer; S1: zeolite amendment, S2: coconut coir amendment, S3: humic acid amendment, S0: no amendment. ANOVA: analysis of variance, S: soil amendment, N: nitrogen fertilizer, S × N: interaction effect between the soil amendment and nitrogen fertilizer, *: *p* < 0.05, **: *p* < 0.01, ***: *p* < 0.001, ns: *p* > 0.05, different lowercase letters represent significant differences between different nitrogen fertilizers under the same soil amendment, different uppercase letters represent significant differences between different soil amendments.

**Table 2:** Effects of different types of soil amendments and nitrogen sources on plant height at different growth stages under salt stress.

Soil Amendments	Nitrogen	Mid-Tillering	Panicle Initiation	Heading	Maturation
S1	N1	67.3 ± 2.1a	92.1 ± 0.8a	109.8 ± 0.7a	112.0 ± 1.7a
	N2	67.1 ± 1.0a	93.3 ± 1.5a	112.3 ± 2.1a	114.7 ± 2.3a
	N0	62.8 ± 1.8b	84.8 ± 2.6b	106.9 ± 0.8b	102.2 ± 1.7b
	Mean	65.8 ± 1.1A	90.1 ± 0.6A	109.7 ± 0.9A	109.6 ± 1.0A
S2	N1	63.3 ± 1.2a	89.1 ± 1.0a	113.3 ± 1.5a	108.8 ± 1.6a
	N2	64.3 ± 2.1a	91.0 ± 2.7a	113.0 ± 0.3a	110.9 ± 2.2a
	N0	60.2 ± 1.0b	81.3 ± 0.6b	104.2 ± 1.7b	102.7 ± 1.5b
	Mean	62.6 ± 0.8C	87.2 ± 0.9B	110.2 ± 0.6A	107.8 ± 1.1AB
S3	N1	64.6 ± 1.4a	86.9 ± 1.7b	109.9 ± 2.2ab	107.8 ± 1.6a
	N2	66.0 ± 1.7a	91.0 ± 1.7a	111.4 ± 1.7a	110.9 ± 2.2a
	N0	60.7 ± 1.2b	78.8 ± 1.6c	107.8 ± 1.6b	102.8 ± 1.5b
	Mean	63.8 ± 1.2B	85.6 ± 1.5B	109.7 ± 0.7A	107.2 ± 1.6AB
S0	N1	64.6 ± 0.5a	89.7 ± 1.5a	108.1 ± 1.8a	108.0 ± 1.5a
	N2	64.5 ± 0.9a	90.4 ± 1.5a	110.3 ± 2.8a	108.4 ± 1.2a
	N0	56.7 ± 2.1b	75.6 ± 1.9b	97.4 ± 2.2b	98.8 ± 1.6b
	Mean	61.9 ± 0.7C	85.2 ± 0.7B	105.3 ± 2.1B	105.0 ± 0.5B
ANOVA	S	*	**	*	*
	N	***	***	***	***
	S × N	ns	*	***	ns

Notes: N1: urea, N2: slow-release urea, N0: no nitrogen fertilizer; S1: zeolite amendment, S2: coconut coir amendment, S3: humic acid amendment, S0: no amendment. ANOVA: analysis of variance, S: soil amendment, N: nitrogen fertilizer, S × N: interaction effect between the soil amendment and nitrogen fertilizer, *: *p* < 0.05, **: *p* < 0.01, ***: *p* < 0.001, ns: *p* > 0.05. Different lowercase letters represent significant differences between different nitrogen fertilizers under the same soil amendment; different uppercase letters represent significant differences between different soil amendments.

**Table 3:** Effects of different types of soil amendments and nitrogen sources on the leaf area index at different growth stages.

Soil Amendments	Nitrogen	Mid-Tillering	Panicle Initiation	Heading
S1	N1	2.1b	3.4b	3.50b
	N2	2.3a	5.4a	5.19a
	N0	1.4c	2.6c	2.63c
	Mean	1.9A	3.8A	3.77A
S2	N1	1.3a	3.4b	3.7b
	N2	1.3a	4.0a	4.1a
	N0	1.1b	2.5c	2.5c
	Mean	1.3B	3.3C	3.5C
S3	N1	1.5b	3.6b	3.9b
	N2	1.6a	4.1a	4.4a
	N0	1.0c	2.4c	2.5c
	Mean	1.4BC	3.4B	3.6B
S0	N1	1.3b	3.4b	3.8a
	N2	1.4a	3.7a	3.7a
	N0	0.9c	1.4c	1.8b
	Mean	1.2C	2.8D	3.1D
ANOVA	S	***	***	***
	N	***	***	***
	S × N	***	***	***

Notes: N1: urea, N2: slow-release urea, N0: no nitrogen fertilizer; S1: zeolite amendment, S2: coconut coir amendment, S3: humic acid amendment, S0: no amendment. ANOVA: analysis of variance, S: soil amendment, N: nitrogen fertilizer, S × N: interaction effect between the soil amendment and nitrogen fertilizer, ***: *p* < 0.001, ns: *p* > 0.05. Different lowercase letters indicate the significant differences between nitrogen fertilizers under the same soil amendment; different uppercase letters represent significant differences between different soil amendments.

**Table 4:** Effects of different types of soil amendments and nitrogen sources on SPAD (chlorophyll content) at different growth stages under salt stress.

Soil Amendments	Nitrogen	Mid-Tillering	Panicle Initiation	Heading
S1	N1	43.5 ± 1.3a	43.5 ± 1.2a	45.1 ± 0.3b
	N2	44.7 ± 1.3a	45.1 ± 0.5a	46.4 ± 0.6a
	N0	43.1 ± 0.7a	40.6 ± 0.3b	40.7 ± 0.8c
	Mean	43.8 ± 0.3A	43.1 ± 0.6A	44.1 ± 0.4A
S2	N1	41.7 ± 0.5b	43.2 ± 0.7a	44.4 ± 0.8a
	N2	44.2 ± 0.4a	42.3 ± 0.4a	44.9 ± 0.8a
	N0	40.3 ± 0.8b	40.5 ± 0.8b	42.5 ± 0.5b
	Mean	42.1 ± 0.1B	42.0 ± 0.9B	44.0 ± 0.2A
S3	N1	44.2 ± 0.4a	45.2 ± 0.7a	44.9 ± 0.5a
	N2	43.9 ± 0.7a	44.3 ± 0.4a	45.0 ± 0.1a
	N0	41.4 ± 0.4b	39.6 ± 1.0b	41.0 ± 0.6b
	Mean	42.8 ± 0.2B	43.7 ± 0.1A	43.7 ± 0.1A
S0	N1	41.3 ± 1.4a	43.3 ± 0.4a	44.4 ± 0.4a
	N2	42.9 ± 0.5a	41.1 ± 0.9a	43.7 ± 0.8a
	N0	37.6 ± 1.1b	36.1 ± 0.7b	38.0 ± 0.4b
	Mean	40.6 ± 0.6C	40.4 ± 0.6C	42.0 ± 0.2B
ANOVA	S	***	**	**
	N	***	***	***
	S × N	***	***	***

Notes: N1: urea, N2: slow-release urea, N0: no nitrogen fertilizer; S1: zeolite amendment, S2: coconut coir amendment, S3: humic acid amendment, S0: no amendment. ANOVA: analysis of variance, S: soil amendment, N: nitrogen fertilizer, S × N: interaction effect between the soil amendment and nitrogen fertilizer, **: *p* < 0.01, ***: *p* < 0.001, ns: *p* > 0.05. Different lowercase letters represent significant differences between different nitrogen fertilizers under the same soil amendment; different uppercase letters represent significant differences between different soil amendments.

**Table 5:** Effects of different types of soil amendments and nitrogen sources on the canopy interception rate at different growth stages.

Soil Amendments	Nitrogen	Mid-Tillering	Panicle Initiation	Heading
S1	N1	57.7 ± 2.5a	71.7 ± 1.5b	73.3 ± 1.2a
	N2	61.0 ± 1.7a	80.0 ± 1.0a	76.7 ± 0.6a
	N0	45.7 ± 3.1b	65.7 ± 2.1c	49.3 ± 1.2b
	Mean	54.8 ± 1.9A	72.4 ± 1.4A	66.4 ± 0.5A
S2	N1	54.0 ± 4.0a	63.0 ± 3.5a	59.0 ± 1.0ab
	N2	53.3 ± 4.7a	63.7 ± 1.5a	62.0 ± 1.7a
	N0	43.7 ± 2.5b	51.7 ± 1.5b	55.3 ± 3.5b
	Mean	50.3 ± 0.9B	59.4 ± 2.0C	58.8 ± 1.8C
S3	N1	62.0 ± 3.6a	65.3 ± 1.5b	64.7 ± 2.5b
	N2	58.3 ± 6.0a	70.0 ± 1.7a	71.7 ± 2.1a
	N0	44.3 ± 2.1b	61.7 ± 2.9c	53.0 ± 3.0c
	Mean	54.9 ± 1.6A	64.3 ± 1.9B	63.1 ± 1.3B
S0	N1	56.7 ± 2.1a	65.3 ± 1.2b	57.7 ± 2.5b
	N2	57.7 ± 2.9a	68.3 ± 2.3a	63.3 ± 1.5a
	N0	39.3 ± 2.1b	45.0 ± 1.0c	36.3 ± 1.5c
	Mean	51.2 ± 1.4B	59.6 ± 1.3C	52.4 ± 1.7D
ANOVA	S	*	***	***
	N	***	***	***
	S × N	ns	***	***

Notes: N1: urea, N2: slow-release urea, N0: no nitrogen fertilizer; S1: zeolite amendment, S2: coconut coir amendment, S3: humic acid amendment, S0: no amendment. ANOVA: analysis of variance, S: soil amendment, N: nitrogen fertilizer, S × N: interaction effect between the soil amendment and nitrogen fertilizer, *: *p* < 0.05, ***: *p* < 0.001, ns: *p* > 0.05. Different lowercase letters represent significant differences between different nitrogen fertilizers under the same soil amendment, and different uppercase letters represent significant differences between different soil amendments.

**Table 6:** Effects of different types of soil amendments and nitrogen sources on the leaf water contents at different growth stages under salt stress.

Soil Amendments	Nitrogen	Mid-Tillering	Panicle Initiation	Heading
S1	N1	63.8 ± 0.8a	56.2 ± 0.6a	54.6 ± 0.6b
	N2	65.0 ± 0.4a	53.5 ± 0.5b	56.4 ± 0.6a
	N0	63.8 ± 0.8a	54.3 ± 0.7b	52.0 ± 0.9c
	Mean	64.2 ± 0.3A	54.8 ± 0.1B	54.4 ± 0.7A
S2	N1	64.7 ± 0.4a	54.8 ± 0.9b	54.7 ± 0.3a
	N2	62.4 ± 0.3b	57.4 ± 0.3a	55.1 ± 0.1a
	N0	62.4 ± 0.1b	52.1 ± 0.1c	50.7 ± 0.6b
	Mean	63.1 ± 0.1B	54.8 ± 0.4B	53.5 ± 0.3B
S3	N1	62.2 ± 0.2b	56.0 ± 1.0b	54.1 ± 1.1a
	N2	64.8 ± 0.2a	57.6 ± 0.2a	53.2 ± 0.2ab
	N0	61.8 ± 0.4b	54.4 ± 0.7c	52.4 ± 0.4b
	Mean	63.0 ± 0.2B	56.0 ± 0.4A	53.3 ± 0.3B
S0	N1	63.1 ± 0.7a	55.7 ± 0.6b	55.2 ± 1.0a
	N2	62.9 ± 0.6a	56.9 ± 0.4a	55.5 ± 0.5a
	N0	63.1 ± 0.8a	52.1 ± 0.5c	49.9 ± 0.4b
	Mean	63.1 ± 0.2B	54.9 ± 0.3B	53.6 ± 0.5B
ANOVA	S	**	*	**
	N	***	***	***
	S × N	***	***	***

Notes: N1: urea, N2: slow-release urea, N0: no nitrogen fertilizer; S1: zeolite amendment, S2: coconut coir amendment, S3: humic acid amendment, S0: no amendment. ANOVA: analysis of variance, S: soil amendment, N: nitrogen fertilizer, S × N: interaction effect between the soil amendment and nitrogen fertilizer, *: *p* < 0.05, **: *p* < 0.01, ***: *p* < 0.001, ns: *p* > 0.05. Different lowercase letters represent significant differences between different nitrogen fertilizers under the same soil amendment, different uppercase letters represent significant differences between different soil amendments.

## Data Availability

The dataset is available on request from the authors.
